# Radiographic Quality and Periapical Status of Post‐Retained Restorations: A Retrospective Cross‐Sectional Study

**DOI:** 10.1155/ijod/2830642

**Published:** 2026-11-15

**Authors:** Khawlah Albashaireh, Ghada Maghaireh, Maram Jaradat, Zakereyya Albashaireh

**Affiliations:** ^1^ Department of Conservative Dentistry Faculty of Dentistry Jordan University of Science and Technology Irbid Jordan just.edu.jo

**Keywords:** endodontically treated teeth, periapical periodontitis, post and core technique, radiography, root canal obturation, tooth crown

## Abstract

**Introduction and Aims:**

Contemporary data evaluating endodontic, post, coronal and practice‐setting parameters of post‐retained restorations together in a multivariable framework remain limited. This multicentre retrospective cross‐sectional study evaluated the radiographic technical quality and current periapical status of post‐retained restorations and identified factors associated with them.

**Methods:**

Periapical radiographs of 459 post‐retained restorations from 366 patients were assessed across three clinical settings in Jordan (February 2020 to December 2025). Post dimensions and angulation, remaining gutta‐percha (RGP), root canal filling length and density, post and coronal marginal adaptation, secondary caries and periapical status (periapical index [PAI]) were recorded. Restorations were graded on a study‐specific composite scale and dichotomised into satisfactory (Grades 1–2) and unsatisfactory (Grade 3). Associations were modelled using generalised estimating equations (GEEs) clustered by patient, reporting adjusted odds ratios (aORs) with 95% confidence intervals (CIs).

**Results:**

Technical quality was satisfactory in 45.8% of restorations (95% CI: 41.2%–50.3%) and periapical radiolucency (PAR) was present in 31.6% (27.5%–36.0%). Radiolucency rose from 28.0% with adequate filling length to 53.6%, where no filling was radiographically evident (clustered trend *p* = 0.002), and was independently associated with absent filling (aOR = 3.09, 95% CI: 1.26–7.56), overextended filling (3.00, 1.21–7.42) and a post‐to‐crown ratio below 0.5:1 (2.87, 1.44–5.69). Satisfactory quality was independently associated with treatment in university faculty specialty clinics (3.76, 1.95–7.23) and student clinics (2.05, 1.11–3.77) relative to private practice, with passive post‐design (1.89, 1.16–3.08) and with younger patient age. The crude association with operator qualification did not persist after adjustment for post‐design (all *p*  > 0.42).

**Conclusion:**

Fewer than half of the restorations examined met the radiographic quality criteria applied. Quality differed by practice setting, post‐retention design and patient age, whereas PAR was most frequent in teeth with no radiographically detectable root filling. These findings describe associations only.

## 1. Introduction

The restoration of endodontically treated teeth has been extensively studied yet remains a subject of clinical debate [1, 2]. Evaluating the clinical performance of post‐retained restorations relies on both clinical examination and radiographic assessment, with radiography serving as an essential diagnostic adjunct alongside clinical parameters such as the remaining coronal tooth structure, ferrule effect, periodontal support, occlusal loading and restorative material properties [2–4]. Because two‐dimensional periapical radiographs provide visualisation of clinically obscured internal root anatomy and surrounding periradicular structures, they remain indispensable for baseline evaluation and routine clinical follow‐up [5].

Periapical radiography enables clinicians to assess the periapical status and the technical adaptation of intra‐radicular and coronal restorations, although diagnostic sensitivity can be influenced by geometric distortion, technical artefacts or anatomical superimposition [6]. High‐quality periapical radiographs are routinely used to evaluate root canal obturation length and density, post‐design and alignment, coronal marginal adaptation and the health of the periapex and adjacent dentition [7–10]. Such cross‐sectional radiographic evaluations document the technical quality of post‐retained restorations and identify procedural deficiencies associated with apical periodontitis [11]. Specifically, radiographic assessment facilitates the detection of technical shortcomings—including inadequate length or homogeneity of the remaining root canal filling, a gap between the apical end of the post and the residual gutta‐percha, deviation of the post from the long axis of the root canal, and poor adaptation of the post or coronal restoration [12]. Clinical and epidemiological studies have reported significant associations between periapical radiolucency (PAR) and the presence of a post‐to‐gutta‐percha gap [13, 14] as well as inadequate root canal obturation [15, 16].

Despite well‐established endodontic and prosthodontic guidelines, previous audits indicate that ideal technical parameters are achieved in only a minority of clinical cases [7]. Recent investigations confirm that suboptimal technical execution remains common in post‐retained restorations and frequently coexists with periapical pathosis [5]. Technical deficiencies such as insufficient post length, axial deviation and poor post‐adaptation have been documented in studies where PAR was observed in nearly half of evaluated teeth [7, 9], while other regional surveys reported PAR in more than half of assessed post‐retained restorations [8].

While three‐dimensional cone‐beam computed tomography (CBCT) is increasingly utilised for complex endodontic diagnosis, conventional periapical radiography remains the primary imaging modality in daily practice [17]. However, contemporary multicentre studies that simultaneously examine endodontic obturation quality, post geometry, coronal marginal adaptation, periapical status and clinical practice sector within an adjusted multivariable framework remain scarce. Furthermore, because individual technical errors rarely occur in isolation, a structured composite technical quality classification alongside component‐level multivariable modelling is needed to distinguish procedural execution from the biological periapical status. Therefore, this study aimed to evaluate the radiographic technical quality of post‐retained restorations and remaining root canal fillings, to determine the prevalence of associated PAR, and to identify patient‐, operator‐ and treatment‐related variables independently associated with these parameters across multiple clinical settings in Jordan.

## 2. Materials and Methods

### 2.1. Study Design and Ethical Approval

This multicentre retrospective cross‐sectional study was designed and reported in accordance with the Strengthening the Reporting of Observational Studies in Epidemiology (STROBE) guidelines for cross‐sectional studies. The investigation evaluated the radiographic technical quality and periapical status of post‐retained restorations in patients treated between February 2020 and December 2025 across three clinical settings in northern Jordan: the Dental Teaching Centre (student clinics and faculty specialty clinics) of Jordan University of Science and Technology (JUST), the Royal Medical Services, and participating private dental practices. The study protocol received formal ethical approval from the Institutional Review Board of JUST (IRB No. July2025/184‐5), which covered the retrospective analysis of records across the participating centres, and administrative authorisation was obtained from the participating clinical facilities prior to data extraction. All retrospective clinical and radiographic records spanning from February 2020 to December 2025 were accessed and analysed following ethical approval. Given the retrospective analysis of routine diagnostic records, the requirement for individual informed consent was waived, and all records were fully de‐identified prior to analysis.

### 2.2. Sample Selection and Case Identification

All patient records held at the participating settings between February 2020 and December 2025 containing a procedure code for an intraradicular post were retrieved from existing institutional and practice archives; no randomisation, sampling fraction or convenience selection was applied at any stage. The corresponding radiographs were then screened against the eligibility criteria and every tooth meeting them was included. Accompanying dental charts were reviewed to extract patient age, gender, practice sector (university student clinics [Dental Teaching Centre], university faculty/specialty clinics, private dental sector and Royal Medical Services), operator qualification (undergraduate dental students, postgraduate Master’s students, general dental practitioners and specialists), restoration age (<1 year, 1–5 years and >5 years) and post‐fabrication method (prefabricated vs. cast post‐and‐core).

The minimum required sample size was calculated for the primary descriptive objective of estimating the prevalence of satisfactory post‐retained restorations, assuming an expected prevalence of 60%, a 95% confidence level, and 5% precision, which yielded a minimum of 369 restorations. Allowing a 20% margin for excluded or incomplete records, the target sample size was set at 442. From an initial screening of 512 retrieved records logged in institutional registries, 53 were excluded due to substandard image quality, cone‐cuts or anatomical superimposition obscuring the periapex, resulting in a final analytic sample of 459 post‐retained restorations from 366 patients (Figure 1). For the secondary analytical objective of multivariable logistic regression modelling, the 145 PAR events across 15 model parameters provided 9.7 events per variable (EPV), providing adequate statistical stability.

**Figure 1:**
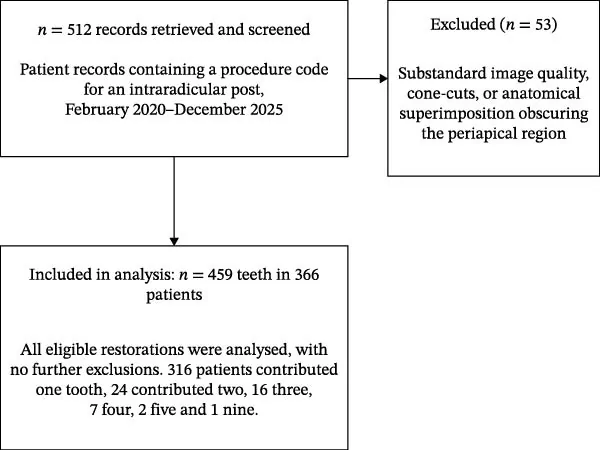
Flow of records through the study. Patient records containing a procedure code for an intraradicular post were retrieved for the period February 2020 to December 2025 and screened against the eligibility criteria; no randomisation or sampling fraction was applied.

### 2.3. Radiographic Assessment and Inclusion Criteria

All radiographic images were anonymised with numerical identifiers prior to evaluation, so that the assessor was blinded to patient identity. Assessment was not otherwise blinded; in particular, blinding to the treatment centre was not feasible because the receptor type (digital photostimulable phosphor plates vs. digitised conventional film) was visually apparent. Radiographs were included if they displayed at least one intraradicular post within a previously root canal‐treated permanent tooth and met diagnostic quality standards—specifically, showing the entire crown and root of interest along with a clearly defined periapical region surrounded by at least 2–3 mm of visible periradicular bone, with optimal contrast and density and free from elongation, foreshortening, cone‐cuts or obscuring artefacts. Radiographs of substandard diagnostic quality or with non‐assessable periapical status were excluded.

### 2.4. Radiographic Acquisition, Calibration and Landmark Definitions

For cases treated at the University Dental Teaching Centre and faculty clinics, digital periapical radiographs were acquired using the long‐cone paralleling technique and photostimulable phosphor (PSP) plates, retrieved from the institutional imaging database, and analysed using the VistaScan Mini View diagnostic system (Dürr Dental, Bietigheim‐Bissingen, Germany); measurements on these images were made in the system’s calibrated software using the same Size 2 receptor dimensions as reference. For cases obtained from external clinics, conventional radiographs were acquired using the long‐cone paralleling technique on standard ANSI Size 2 Kodak Insight film (31 mm × 41 mm; Eastman Kodak Co., Rochester, NY, USA) and digitised using a flatbed scanner with a transparency unit (CanoScan LiDE 700 F, Canon, Tokyo, Japan) at 600 dpi spatial resolution. Prior to performing linear measurements on digitised conventional films, each image was individually calibrated in the diagnostic measurement software against the known physical dimensions of the standard Size 2 film packet (31 mm × 41 mm). Where necessary to improve visualisation of the cemento‐enamel junction (CEJ) or root apex, image brightness and contrast were adjusted by the calibrated examiner using the software’s standardised histogram‐equalisation tool. Adjustment was applied only where required rather than uniformly to all images, was limited to brightness and contrast, and no sharpening, magnification or other image processing was performed.

Radiographs were evaluated for the following anatomical and technical parameters:

1. Post, crown and root dimensions: Crown length was measured along the long axis of the tooth from the most coronal incisal edge or occlusal cusp tip to the mid‐root intersection of a reference line connecting the mesial and distal CEJ landmarks (or the apical crown margin when the CEJ was obscured by the coronal restoration). Root length was measured along the longitudinal root canal axis from the CEJ/restorative margin reference line to the radiographic apex. Post length was measured from the coronal termination of the intraradicular post (at the reference line) to its most apical tip. Because minor differences in projection geometry across imaging modalities can influence absolute linear measurements, post length was additionally expressed as dimensionless ratios relative to crown length (post‐to‐crown ratio) and root length (post‐to‐root ratio).
2. Post‐angulation: Post‐deviation from the root canal axis in the mesio‐distal plane was measured as the acute angle (in degrees) formed between the central longitudinal axis of the post and the central longitudinal axis of the root canal; an angular deviation >2° was recorded as a measurable axial deviation.
3. Apical post‐filling interface: The linear distance (mm) of remaining gutta‐percha (RGP) from the apical end of the post to the apical termination of the root filling was measured in all teeth retaining the apical filling material (*n* = 429). The presence and linear extent (mm) of any radiolucent space (gap) between the apical tip of the post and the coronal aspect of the residual root canal filling were recorded.
4. Root canal filling length and density: Evaluated according to the European Society of Endodontology (ESE) quality guidelines [18, 19]. Length was categorised as adequate (0–2.0 mm short of the radiographic apex), underfilled (>2.0 mm short), overextended (extending beyond the radiographic apex) or absent (no radiographically evident root filling within the canal). Density of the remaining obturation (*n* = 431 teeth with root fillings) was classified as satisfactory (homogeneous radiopacity without visible voids) or poor (detectable voids or laterally under‐condensed ’honeycombed’ appearance).
5. Multi‐rooted teeth and periapical scoring: For multi‐rooted teeth (*n* = 74), clinical protocol typically placed a single post in the widest, straightest canal (palatal canal in maxillary molars or distal canal in mandibular molars); where more than one post was present, only the primary post in the widest canal was analysed to maintain a consistent tooth‐level unit of observation. Periapical status was scored using the 5‐point periapical index (PAI) [20], ranging from 1 (normal periapical structures) to 5 (severe periodontitis with exacerbating features). For multi‐rooted teeth, the highest (worst) root‐specific PAI score among all roots was assigned to the tooth. In accordance with standard epidemiological conventions, PAI scores were dichotomised into healthy periapex (PAI 1–2) and PAR indicative of apical periodontitis (PAI ≥ 3).
6. Adaptation and supplemental findings: Post‐to‐canal wall adaptation was recorded as unsatisfactory when a distinct radiolucent gap was visible laterally between the post surface and the inner root canal walls. Coronal marginal adaptation was classified as unsatisfactory in the presence of visible open margins, overhangs or defective adaptation at the crown‐to‐tooth interface. Pathological and structural findings—including secondary caries at the restorative margins, internal or external root resorption, root perforations, root fractures and post fractures—were recorded separately.

### 2.5. Examiner Calibration and Reliability

Prior to evaluating the study dataset, a structured training and calibration protocol was conducted using a reference set of 50 periapical radiographs encompassing the full range of technical parameters and PAI scores [20]. A senior specialist trained and calibrated a single primary clinician on all anatomical reference points, software calibration procedures, linear and angular measurements, PAI reference radiographs and technical quality criteria. Following calibration, all 459 radiographs in the study sample were evaluated solely by this single calibrated clinician to eliminate inter‐examiner variability across the entire dataset, with any borderline cases resolved by consensus with the senior specialist. Intra‐examiner reliability was assessed by re‐evaluating the random subsample of 50 radiographs after a 2‐week interval, demonstrating an absolute agreement of 95% and substantial agreement (Cohen’s *κ* = 0.77) for the dichotomous overall radiographic technical quality classification.

### 2.6. Composite Technical Quality Grading Scale

To evaluate overall procedural execution without conflating technical quality with biological periapical disease, a study‐specific three‐grade composite technical quality classification was synthesised from ESE endodontic guidelines [18, 19] and established prosthodontic benchmarks [1] [7, 10, 21] [22–27]. Crucially, biological and pathological findings (PAR [PAI ≥ 3] secondary caries and internal or external root resorption) were excluded from the composite technical quality score so that the association between procedural parameters and periapical status could be analysed independently without circularity. The composite grade was derived algorithmically from the individually recorded parameters using a ‘worst‐score‐counts’ rule across three categories.

#### 2.6.1. Grade 1 (Good/Ideal)

All core endodontic, restorative, and dimensional parameters met ideal standards, including root canal filling terminating 0–2.0 mm short of the apex with homogeneous density, RGP length ≥ 4.0 mm, post‐to‐crown ratio ≥ 1:1, axial post alignment without deviation, optimal post and coronal marginal adaptation and absence of mechanical/iatrogenic complications.

#### 2.6.2. Grade 2 (Acceptable)

Restorations free of major endodontic, coronal‐seal or mechanical failures, exhibiting minor dimensional deviations or minor lateral post adaptation discrepancies not requiring clinical intervention (e.g., post‐to‐crown ratio between 0.5:1 and <1:1, minor lateral post‐space discrepancy with intact coronal and apical seals, RGP length ≥ 3.0 mm, minor axial deviation ≤ 10° or root filling termination within 0–2.0 mm of the apex or ≤ 1.0 mm apical extrusion).

#### 2.6.3. Grade 3 (Poor/Unsatisfactory)

Presence of one or more major technical or mechanical deficiencies compromising the apical seal coronal seal, or structural integrity—specifically: (i) root canal filling >2.0 mm short of the apex, overextended >1.0 mm beyond the apex or absent; (ii) defective coronal marginal adaptation; or (iii) mechanical or iatrogenic structural failure (root fracture, post‐fracture or root perforation). In addition, a strict sensitivity classification was evaluated in which any minor geometric, adaptation or density deviation (post‐to‐crown ratio <0.5:1, RGP <3.0 mm, post‐deviation >10°, post‐to‐canal wall gap post‐to‐filling gap, or root filling voids) also triggered Grade 3.

For statistical analysis, Grades 1 and 2 were dichotomised as ‘satisfactory’ technical quality, whereas Grade 3 was classified as ‘unsatisfactory’. The 3.0 mm threshold for RGP was prespecified on the basis of radiographic evidence that posted roots retaining less than 3 mm of root filling show a significantly higher frequency of PAR [24], supported by in vitro findings that reduction of the remaining filling to this length yields an unpredictable seal [23].

### 2.7. Data Analysis

Data management and statistical analyses were performed using IBM SPSS Statistics (version 28.0; IBM Corp., Armonk, NY, USA) and Python (statsmodels version 0.14). Descriptive statistics were summarised as frequencies and percentages with 95% Wilson confidence intervals (CIs) for categorical variables, and as means ± standard deviations (SDs) alongside medians and interquartile ranges (IQRs) for continuous variables. Unadjusted associations between categorical variables were initially explored using Pearson’s chi‐square (*χ* ^2^) test, or the Fisher–Freeman–Halton exact test when expected cell frequencies were less than five. Patient age was analysed both as a continuous predictor and in conventional decade bands, the latter retained for comparability with earlier audits of post‐retained restorations, and estimates from both specifications are reported. Age of restoration was recorded in the source records as intervals (<1 year, 1–5 years, >5 years) rather than as exact dates and could therefore only be analysed categorically.

Because 50 of the 366 patients (13.7%) contributed more than one post‐retained restoration, tooth‐level observations were clustered within patients. To account for intra‐patient clustering and control for confounding, univariable and multivariable binary logistic regression models were fitted using generalised estimating equations (GEEs) with a binomial distribution, logit link function, exchangeable working correlation matrix and robust (Huber–White sandwich) standard errors with unique patient identifier specified as the clustering variable. Linear trend across ordered root filling length categories was tested using a clustered GEE trend model. For periapical status (PAI ≥ 3 vs. PAI 1–2), a multivariable GEE model was fitted incorporating root canal filling length, obturation density, RGP length (<3 mm), post‐root filling gap, post‐to‐canal wall adaptation, coronal marginal adaptation, secondary caries, post‐to‐crown ratio, post‐retention design, tooth type and restoration age. For overall radiographic technical quality (satisfactory vs. unsatisfactory), collinearity between practice sector and operator qualification was evaluated using Cramér’s *V*; because these two variables exhibited strong structural collinearity (*χ* ^2^ = 605.75, *p*  < 0.001, Cramér’s *V* = 0.663), two separate multivariable GEE models were fitted: Model A (evaluating practice sector alongside patient age, gender, restoration age and post design) and Model B (evaluating operator qualification alongside patient age, gender, restoration age and post design). In both multivariable models, patient age was entered as a continuous predictor (years); the conventional decade bands are retained for descriptive tabulation and were analysed in a parallel categorical specification. Results were expressed as unadjusted odds ratios (ORs) and adjusted odds ratios (aORs) with 95% CIs, with statistical significance set at two‐tailed *p*  < 0.05.

## 3. Results

### 3.1. Sample Characteristics and Patient‐Level Clustering

A total of 459 post‐retained restorations from 366 unique patients met the inclusion criteria. Regarding intra‐patient clustering, 316 patients (86.3%) contributed a single restoration, 24 patients (6.6%) contributed two restorations and 26 patients (7.1%) contributed three or more restorations (specifically, 16 patients with three teeth, 7 with four teeth, 2 with five teeth and 1 with nine teeth). At the patient level (*n* = 366), the mean age was 39.6 ± 14.0 years (median: 39.0, IQR: 21.0, range: 14–75 years), and 211 patients (57.7%) were female, while 155 (42.3%) were male. At the tooth level (*n* = 459), the mean patient age was 40.2 ± 13.9 years (median: 40.0, IQR: 21.0), with 248 restorations (54.0%) in female patients and 211 (46.0%) in male patients. Post‐retained restorations were placed more frequently in maxillary teeth (68.6%; *n* = 315) than in mandibular teeth (31.4%; *n* = 144), and premolars were the most commonly restored tooth type (46.8%; *n* = 215), followed by anterior teeth (37.0%; *n* = 170) and molars (16.1%; *n* = 74). The distribution by dental arch and tooth type is presented in Table 1.

**Table 1:** Distribution of post‐retained restorations by dental arch and tooth type (*n* = 459 teeth in 366 patients).

Arch	Anterior, *n* (%)	Premolar, *n* (%)	Molar, *n* (%)	Total, *n* (%)
Maxillary	151 (32.9)	138 (30.1)	26 (5.7)	315 (68.6)
Mandibular	19 (4.1)	77 (16.8)	48 (10.5)	144 (31.4)
Total	170 (37.0)	215 (46.8)	74 (16.1)	459 (100.0)

### 3.2. Post Characteristics and Dimensions

The radiographic distributions of post shape, retention design and fabrication method across the 459 restorations are shown in Figure 2. Tapered posts accounted for 79.5% (*n* = 365) of cases, whereas 20.5% (*n* = 94) were parallel‐sided. Regarding retention mode, passive posts represented 57.1% (*n* = 262) of cases and active screw‐type posts accounted for 42.9% (*n* = 197). Prefabricated posts were identified in 73.4% (*n* = 337) of restored teeth, while cast post‐and‐core restorations accounted for the remaining 26.6% (*n* = 122).

**Figure 2:**
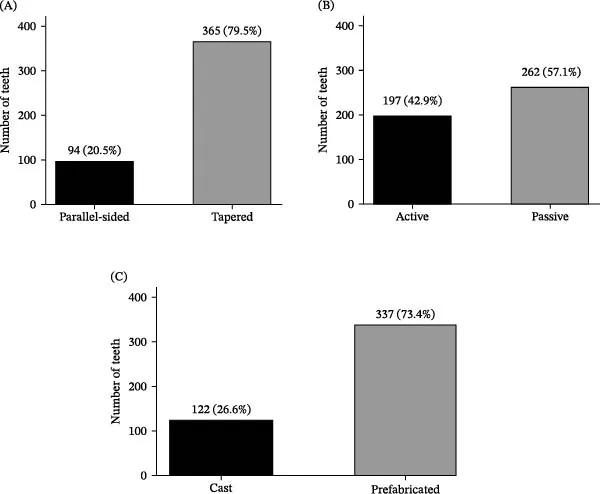
Radiographic characteristics of posts among the evaluated teeth (*n* = 459). (A) Post shape: parallel‐sided (*n* = 94; 20.5%) and tapered (*n* = 365; 79.5%). (B) Mode of retention: active (*n* = 197; 42.9%) and passive (*n* = 262; 57.1%). (C) Fabrication method: cast (*n* = 122; 26.6%) and prefabricated (*n* = 337; 73.4%).

Across all 459 teeth, absolute crown length averaged 7.4 ± 1.3 mm (median: 7.4, IQR: 1.8, range: 3.8–11.5 mm), root length averaged 13.8 ± 2.6 mm (median: 13.8, IQR: 3.4, range: 3.3–22.0 mm) and post length averaged 6.4 ± 2.6 mm (median: 6.0, IQR: 3.5, range: 1.3–17.6 mm). The mean post‐to‐crown length ratio was 0.90 ± 0.41 (median: 0.85, IQR: 0.52, range: 0.18–2.45). Posts measuring between 0.5:1 and <1:1 of the crown length were most prevalent (49.7%; *n* = 228), followed by posts equal to or exceeding crown length (≥1:1; 33.8%, 95% CI: 29.6%–38.2%, *n* = 155) and those shorter than half the crown length (<0.5:1; 16.6%, *n* = 76; Table 2). The mean post‐to‐root length ratio was 0.47 ± 0.23 (median: 0.45, IQR: 0.23). Most posts (61.2%; *n* = 281) had a post‐to‐root ratio below 0.5:1, while 25.3% (*n* = 116) ranged between 0.5:1 and 0.65:1 and 13.5% (*n* = 62) exceeded 0.65:1 (Figure 3).

**Figure 3:**
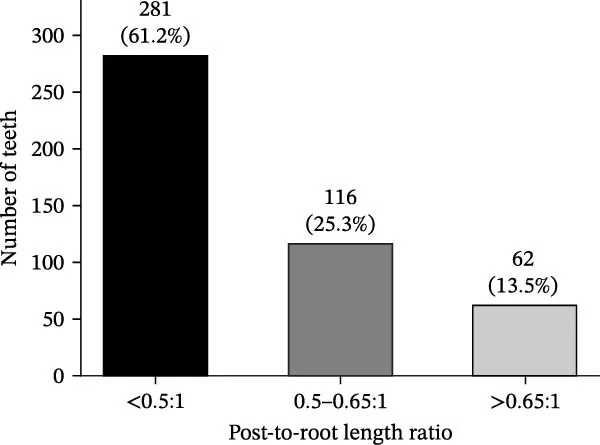
Distribution of post‐to‐root length ratio (*n* = 459): <0.5:1 (*n* = 281; 61.2%), 0.5–0.65:1 (*n* = 116; 25.3%) and >0.65:1 (*n* = 62; 13.5%).

**Table 2:** Univariable and multivariable patient‐clustered GEE analysis of endodontic, post and coronal factors associated with periapical radiolucency (PAI ≥ 3; *n* = 459 teeth in 366 patients).

Variable/category	Total (*n*)	Healthy periapex, *n* (%)	Periapical radiolucency, *n* (% [95% CI])	Unadjusted GEE OR (95% CI)	Unadjusted *p*‐Value ^∗^	Adjusted GEE aOR (95% CI)^a^	Adjusted *p*‐Value^a^
Root canal filling length					**0.022**		
0–2 mm short of apex (Ref)	318	229 (72.0)	89 (28.0 [23.3–33.2])	1.00	—	1.00	—
>2 mm short of apex	88	58 (65.9)	30 (34.1 [25.0–44.5])	1.30 (0.78–2.17)	0.308	0.81 (0.44–1.50)	0.502
Beyond apex (overextended)	25	14 (56.0)	11 (44.0 [26.7–62.9])	2.13 (0.93–4.88)	0.075	3.00 (1.21–7.42)	0.018
No radiographic root filling	28	13 (46.4)	15 (53.6 [35.8–70.5])	2.81 (1.28–6.17)	0.010	3.09 (1.26–7.56)	0.014
Obturation density^b^					**0.006**		
Satisfactory (Ref)	261	196 (75.1)	65 (24.9 [20.0–30.5])	1.00	—	1.00	—
Poor (voids present)	170	105 (61.8)	65 (38.2 [31.3–45.7])	1.82 (1.19–2.78)	0.006	1.44 (0.89–2.32)	0.136
Remaining GP length (harmonised)^c^					**0.027**		
>5.0 mm (Ref)	278	196 (70.5)	82 (29.5 [24.4–35.1])	1.00	—	1.00 (≥3 mm)	—
3.0–5.0 mm (4–5 mm: 24/81 [29.6%])	124	89 (71.8)	35 (28.2 [21.1–36.7])	0.98 (0.62–1.56)	0.944	—	—
<3.0 mm (<4 mm: 23/70 [32.9%])	27	15 (55.6)	12 (44.4 [27.6–62.7])	2.02 (0.88–4.66)	0.098	2.05 (0.86–4.89)	0.105
No GP apical to post	30	14 (46.7)	16 (53.3 [36.1–69.8])	2.69 (1.23–5.86)	0.013	—	—
Post–root filling gap^b^					**0.306**		
Absent (Ref)	288	207 (71.9)	81 (28.1 [23.2–33.6])	1.00	—	1.00	—
Present (0.2–5.4 mm)	142	94 (66.2)	48 (33.8 [26.5–41.9])	1.26 (0.81–1.96)	0.306	1.20 (0.73–1.95)	0.475
Post adaptation to canal walls					**0.073**		
Satisfactory (Ref)	360	255 (70.8)	105 (29.2 [24.7–34.1])	1.00	—	1.00	—
Unsatisfactory	99	59 (59.6)	40 (40.4 [31.3–50.3])	1.57 (0.96–2.57)	0.073	1.05 (0.62–1.80)	0.853
Coronal marginal adaptation	—	—	—	—	**0.010**	—	—
Satisfactory (Ref)	290	212 (73.1)	78 (26.9 [22.1–32.3])	1.00	—	1.00	—
Unsatisfactory	169	102 (60.4)	67 (39.6 [32.6–47.2])	1.74 (1.14–2.66)	0.010	1.36 (0.84–2.21)	0.217
Secondary caries on restored tooth					**0.006**		
Absent (Ref)	405	286 (70.6)	119 (29.4 [25.2–34.0])	1.00	—	1.00	—
Present	54	28 (51.9)	26 (48.1 [35.4–61.1])	2.22 (1.26–3.91)	0.006	1.89 (0.99–3.62)	0.054
Post‐to‐crown length ratio					**<0.001**		
≥1.0:1 (Ref)	155	122 (78.7)	33 (21.3 [15.6–28.4])	1.00	—	1.00	—
0.5–<1.0:1	228	151 (66.2)	77 (33.8 [27.9–40.1])	1.85 (1.18–2.91)	0.008	1.90 (1.11–3.27)	0.020
<0.5:1	76	41 (53.9)	35 (46.1 [35.3–57.2])	3.07 (1.71–5.50)	<0.001	2.87 (1.44–5.69)	0.003
Post retention design					**0.127**		
Passive (Ref)	262	188 (71.8)	74 (28.2 [23.1–34.0])	1.00	—	1.00	—
Active (screw‐type)	197	126 (64.0)	71 (36.0 [29.7–43.0])	1.38 (0.91–2.09)	0.127	1.04 (0.62–1.74)	0.890
Tooth type					**0.081**		
Molar (Ref)	74	59 (79.7)	15 (20.3 [12.7–30.8])	1.00	—	1.00	—
Premolar	215	145 (67.4)	70 (32.6 [26.6–39.1])	1.84 (1.01–3.38)	0.048	2.16 (1.10–4.25)	0.025
Anterior	170	110 (64.7)	60 (35.3 [28.5–42.7])	2.07 (1.08–3.96)	0.028	2.41 (1.15–5.06)	0.020
Age of restoration					**0.280**		
1–5 years (Ref)	308	219 (71.1)	89 (28.9 [24.1–34.2])	1.00	—	1.00	—
<1 year	96	61 (63.5)	35 (36.5 [27.5–46.4])	1.37 (0.84–2.23)	0.212	1.16 (0.68–1.98)	0.598
>5 years	55	34 (61.8)	21 (38.2 [26.5–51.4])	1.47 (0.81–2.69)	0.210	1.05 (0.55–1.99)	0.881
Total	459	314 (68.4)	145 (31.6 [27.5–36.0])	—	—	—	—

*Note*: Bold type identifies the variable‐level summary row for each variable and does not denote statistical significance.

Abbreviations: aOR, adjusted odds ratio; CI, confidence interval; GEE, generalised estimating equation; GP, gutta‐percha; OR, odds ratio; PAI, periapical index.

^a^Adjusted odds ratios derive from a single multivariable GEE model (binomial family, logit link, exchangeable working correlation, robust standard errors, patient as the clustering variable) containing root canal filling length, obturation density, remaining gutta‐percha length, post–root filling gap, post adaptation to canal walls, coronal marginal adaptation, secondary caries, post‐to‐crown length ratio, post retention design, tooth type and age of restoration.

^b^Denominator excludes teeth in which the variable is undefined because no root canal filling was radiographically evident (obturation density, *n* = 431; post‐root filling gap, *n* = 430); in the multivariable model these variables were assigned the reference category for those teeth so that all 459 teeth, including the 28 with no radiographically evident root filling, were retained.

^c^Categories harmonised to the prespecified 3.0 mm threshold; values in parentheses give the corresponding figures for the 4–5 mm and <4 mm bands. Remaining gutta‐percha entered the multivariable model as a binary variable (<3.0 mm vs. ≥3.0 mm); dashes denote categories not separately estimated.

^∗^Unadjusted *p*‐value from the patient‐clustered GEE Wald test for the overall effect of each variable.

### 3.3. RGP and Root Canal Filling Quality

Among the 429 teeth retaining gutta‐percha apical to the post tip, the mean length of RGP was 6.2 ± 2.2 mm (median: 6.0, IQR: 2.9, range: 0.5–12.7 mm). Using the harmonised grading thresholds (Table 2), 60.6% (*n* = 278) of all 459 teeth retained >5.0 mm of RGP, 27.0% (*n* = 124) retained 3.0–5.0 mm (including 17.6% [*n* = 81] with 4.0–5.0 mm and 9.4% [*n* = 43] with 3.0–3.9 mm) and 5.9% (*n* = 27) retained <3.0 mm (making 15.3% [*n* = 70] with <4.0 mm in total). No radiographic evidence of gutta‐percha apical to the post was observed in 6.5% (*n* = 30) of cases; this group comprised 28 teeth (6.1%) with no radiographic evidence of root canal filling anywhere within the canal and two additional teeth (0.4%) in which root canal filling material was present but the post extended to the apical termination of the filling, leaving 0 mm of RGP apical to the post.

Regarding overall root canal filling length (*n* = 459), 69.3% (95% CI: 64.9%–73.3%; *n* = 318) of teeth had fillings terminating within the acceptable range (0–2.0 mm short of the radiographic apex), 19.2% (*n* = 88) were underfilled (>2.0 mm short), 5.4% (*n* = 25) were overextended beyond the apex and 6.1% (*n* = 28) had no radiographic root filling within the canal (Table 2). Among the 431 teeth with a root canal filling present, obturation density was satisfactory in 60.6% (261/431; representing 56.9% of the total 459‐tooth sample) and poor (visible voids) in 39.4% (170/431; 37.0% of the total sample).

### 3.4. Post‐Deviation, Adaptation, Coronal Margins and Supplemental Findings

Deviation of the post from the long axis of the root canal was identified in 57 teeth (12.4%), with measurable quantitative angulation recorded in 50 teeth (mean deviation angle: 10.4° ± 6.1°, median: 9.4°, IQR: 8.2°, range: 2.4°–34.5°; or 9.9° ± 5.0° across cases ≤ 20.0°), while 7 cases exhibited minor qualitative angulation without numerical logging. Radiographic evidence of root perforation was detected in 1.3% (*n* = 6) of teeth.

A radiolucent gap between the apical end of the post and the coronal aspect of the residual root filling was present in 30.9% (*n* = 142/459; or 32.9% of the 431 teeth with root filling material). A linear measurement of the gap was available for 138 of these 142 teeth (mean 1.34 ± 1.02 mm, median: 1.00, IQR: 0.98, range: 0.20–5.40 mm); the gap extent was not recorded in the remaining four. Lateral adaptation of the post to the canal walls was unsatisfactory in 21.6% (*n* = 99) of teeth. Coronal marginal adaptation was satisfactory in 63.2% (*n* = 290) of restorations and unsatisfactory in 36.8% (*n* = 169). Secondary caries was detected radiographically on 11.8% (*n* = 54) of post‐restored teeth and 18.7% (*n* = 86) of adjacent teeth. External root resorption was observed in 4.1% (*n* = 19) of teeth, internal root resorption in 0.9% (*n* = 4), root fracture in 2.0% (*n* = 9) and post‐fracture in 0.7% (*n* = 3).

### 3.5. Periapical Status and Multivariable GEE Analysis of Associated Technical Factors

Overall, PAR (PAI ≥ 3) was present in 31.6% (95% CI: 27.5%–36.0%; *n* = 145/459) of evaluated teeth, whereas 68.4% (*n* = 314) exhibited a healthy periapex (PAI 1–2). Univariable and multivariable patient‐clustered GEE analyses of technical and restorative factors associated with PAR are presented in Table 2. Across root canal filling length categories, PAR prevalence rose progressively from 28.0% (95% CI: 23.3%–33.2%) in teeth with adequate obturation length (0–2 mm short) to 34.1% (95% CI: 25.0%–44.5%) in underfilled teeth (>2 mm short), 44.0% (95% CI: 26.7%–62.9%) in overextended fillings, and 53.6% (95% CI: 35.8%–70.5%) in teeth with no radiographically evident root filling (unadjusted *χ* ^2^ = 10.206, *p* = 0.017; GEE linear trend OR per category ste*p* = 1.41, 95% CI: 1.13–1.75, *p* = 0.002). Notably, among the 145 teeth presenting with PAR, 61.4% (89/145) exhibited an adequate root filling length (0–2 mm short of the apex).

Regarding RGP apical to the post, PAR was observed in 53.3% (16/30) of teeth with no apical gutta‐percha compared with 44.4% (12/27) of those with <3.0 mm, 28.2% (35/124) of those with 3.0–5.0 mm (29.6% [24/81] for 4.0–5.0 mm and 32.9% [23/70] for <4.0 mm overall), and 29.5% (82/278) of those with >5.0 mm (harmonised *χ* ^2^ = 9.841, *p* = 0.020; clustered GEE *p* = 0.027). When the 30 teeth lacking apical gutta‐percha were excluded (*n* = 429), PAR prevalence did not differ significantly across the categories retaining gutta‐percha (*χ* ^2^ = 0.310, *p* = 0.857 for >5, 4–5 and <4 mm; *χ* ^2^ = 2.897, *p* = 0.235 for >5, 3–5 and <3 mm; similarly, across overall root canal filling categories among teeth with filling present [0–2 mm, >2 mm and beyond apex], *χ* ^2^ = 3.631, *p* = 0.163).

In univariable clustered GEE analyses (Table 2), higher odds of PAR were significantly associated with absent root filling (OR = 2.81, *p* = 0.010), poor obturation density (38.2% vs. 24.9%; OR = 1.82, *p* = 0.006), unsatisfactory coronal marginal adaptation (39.6% vs. 26.9%; OR = 1.74, *p* = 0.010), secondary caries (48.1% vs. 29.4%; OR = 2.22, *p* = 0.006) and lower post‐to‐crown length ratios (<0.5:1 vs. ≥1:1: 46.1% vs. 21.3%, OR = 3.07, *p*  < 0.001; 0.5–<1:1 vs. ≥1:1: 33.8% vs. 21.3%, OR = 1.85, *p* = 0.008). In the multivariable GEE model (Table 2), the factors that remained independently associated with higher odds of PAR were: no radiographic root filling (aOR = 3.09, 95% CI: 1.26–7.56, *p* = 0.014), overextended root filling beyond the apex (aOR = 3.00, 95% CI: 1.21–7.42, *p* = 0.018), post‐to‐crown length ratio <0.5:1 (aOR = 2.87, 95% CI: 1.44–5.69, *p* = 0.003) and 0.5–<1:1 (aOR = 1.90, 95% CI: 1.11–3.27, *p* = 0.020) relative to ≥ 1:1, and anterior (aOR = 2.41, 95% CI: 1.15–5.06, *p* = 0.020) or premolar (aOR = 2.16, 95% CI: 1.10–4.25, *p* = 0.025) tooth types compared with molars. Secondary caries showed a borderline independent association (aOR = 1.89, 95% CI: 0.99–3.62, *p* = 0.054), whereas the unadjusted associations with obturation density (*p* = 0.136), coronal marginal adaptation (*p* = 0.217), post adaptation (*p* = 0.853) and post‐to‐filling gap (*p* = 0.475) were attenuated after adjustment.

### 3.6. Composite Radiographic Technical Quality and Multivariable GEE Analysis

Based on the primary composite technical quality classification, 45.8% (95% CI: 41.2%–50.3%; *n* = 210/459) of restorations were classified as satisfactory (Grades 1–2), while 54.2% (*n* = 249) were classified as unsatisfactory (Grade 3). Under the strict sensitivity classification requiring simultaneous fulfilment of all dimensional, density and adaptation criteria, 25.3% (95% CI: 21.5%–29.4%; *n* = 116/459) of teeth were classified as satisfactory. Before reclassifying the two isolated post‐fracture cases into Grade 3, the satisfactory proportion was 46.2% (*n* = 212/459). Restorations classified as technically unsatisfactory exhibited a significantly higher prevalence of PAR than satisfactory restorations (38.6% [96/249] vs. 23.3% [49/210], unadjusted *χ* ^2^ = 12.213, *p*  < 0.001; GEE OR = 2.03, 95% CI: 1.33–3.10, *p* = 0.001).

Univariable and multivariable GEE analyses of patient, operator and restoration characteristics associated with satisfactory technical quality are detailed in Table 3. In unadjusted analyses, technical quality was significantly associated with patient age group (*χ* ^2^ = 13.866, *p* = 0.008; clustered GEE Wald *p* = 0.024), practice sector (*χ* ^2^ = 31.718, Fisher–Freeman–Halton exact *p*  < 0.001; clustered GEE Wald *p*  < 0.001), operator qualification (*χ* ^2^ = 11.246, *p* = 0.010; clustered GEE Wald *p* = 0.025), post‐retention design (*χ* ^2^ = 30.56, *p*  < 0.001; 56.9% satisfactory for passive vs. 31.0% for active posts) and age of restoration (*χ* ^2^ = 6.353, *p* = 0.042; clustered GEE Wald *p* = 0.022), but not patient gender (*χ* ^2^ = 0.576, *p* = 0.448; GEE *p* = 0.395). Cross‐tabulation revealed that passive posts predominated in undergraduate cases (95.9%) and Master’s student cases (76.0%), whereas active posts predominated in general practitioner cases (78.4%).

**Table 3:** Univariable and multivariable patient‐clustered GEE analysis of patient, operator and restoration characteristics associated with satisfactory radiographic technical quality (*n* = 459 teeth in 366 patients).

Variable/category	Total (*n*)	Unsatisfactory, *n* (%)	Satisfactory, *n* (% [95% CI])	Pearson *χ* ^2^	Unadjusted *χ* ^2^ *p*‐Value ^∗^	Unadjusted GEE OR (95% CI; *p*‐Value)^a^	Adjusted GEE Model A aOR (95% CI; *p*‐Value)^b^	Adjusted GEE Model B aOR (95% CI; *p*‐Value)^c^
Patient age group (years)‖	—	—	—	**13.866**	**0.008**	**Wald** **p** = 0.024	**0.97 (0.96–0.99; 0.001) per year**	**0.98 (0.96–0.99; 0.006) per year**
18–28	114	45 (39.5)	69 (60.5 [51.4–69.0])	—	—	2.36 (1.33–4.18; 0.003)	—	—
29–39	111	64 (57.7)	47 (42.3 [33.6–51.6])	—	—	1.13 (0.64–2.01; 0.665)	—	—
40–50 (Ref)	120	74 (61.7)	46 (38.3 [30.1–47.3])	—	—	1.00	—	—
51–61	82	48 (58.5)	34 (41.5 [31.4–52.3])	—	—	1.09 (0.59–2.02; 0.785)	—	—
>61	32	18 (56.2)	14 (43.8 [28.2–60.7])	—	—	1.19 (0.51–2.79; 0.686)	—	—
Gender‖	—	—	—	**0.576**	**0.448**	**Wald** **p** = 0.357	—	—
Male (Ref)	211	119 (56.4)	92 (43.6 [37.1–50.4])	—	—	1.00	1.00	1.00
Female	248	130 (52.4)	118 (47.6 [41.4–53.8])	—	—	1.21 (0.81–1.80; 0.357)	1.35 (0.88–2.08; 0.170)	1.27 (0.84–1.94; 0.261)
Practice sector	—	—	—	**31.718**	**<0.001¶**	**Wald** **p** < 0.001	—	**(Omitted: collinear)**
Private sector (Ref)	196	136 (69.4)	60 (30.6 [24.6–37.4])	—	—	1.00	1.00	—
University student clinics (DTC)	127	56 (44.1)	71 (55.9 [47.2–64.2])	—	—	2.76 (1.68–4.54; <0.001)	2.05 (1.11–3.77; 0.022)	—
University faculty specialty clinic	129	54 (41.9)	75 (58.1 [49.5–66.3])	—	—	3.05 (1.81–5.14; <0.001)	3.76 (1.95–7.23; <0.001)	—
Royal Medical Services	7	3 (42.9)	4 (57.1 [25.0–84.2])	—	—	3.02 (0.66–13.92; 0.156)	2.47 (0.37–16.54; 0.351)	—
Post retention design	—	—	—	**30.560**	**<0.001**	**Wald** **p** < 0.001	—	—
Active screw‐type (Ref)	197	136 (69.0)	61 (31.0 [24.9–37.8])	—	—	1.00	1.00	1.00
Passive design	262	113 (43.1)	149 (56.9 [50.8–62.8])	—	—	2.77 (1.84–4.15; <0.001)	1.89 (1.16–3.08; 0.011)	2.86 (1.78–4.61; <0.001)
Operator qualification	—	—	—	**11.246**	**0.010**	**Wald** **p** = 0.025	**(Omitted: collinear)**	—
General practitioner (Ref)	116	76 (65.5)	40 (34.5 [26.5–43.5])	—	—	1.00	—	1.00
Specialist	219	118 (53.9)	101 (46.1 [39.6–52.7])	—	—	1.46 (0.90–2.37; 0.125)	—	1.18 (0.68–2.05; 0.552)
Postgraduate student	75	35 (46.7)	40 (53.3 [42.2–64.2])	—	—	1.86 (0.93–3.71; 0.077)	—	1.36 (0.64–2.93; 0.425)
Undergraduate student	49	20 (40.8)	29 (59.2 [45.2–71.8])	—	—	2.76 (1.39–5.47; 0.004)	—	0.98 (0.41–2.32; 0.961)
Age of restoration	—	—	—	**6.353**	**0.042**	**Wald** **p** = 0.022	—	—
>5 years (Ref)	55	36 (65.5)	19 (34.5 [23.4–47.7])	—	—	1.00	1.00	1.00
1–5 years	308	170 (55.2)	138 (44.8 [39.4–50.4])	—	—	1.29 (0.70–2.38; 0.412)	0.65 (0.31–1.35; 0.249)	1.05 (0.53–2.08; 0.888)
<1 year	96	43 (44.8)	53 (55.2 [45.3–64.8])	—	—	2.34 (1.16–4.70; 0.018)	1.26 (0.57–2.81; 0.567)	1.65 (0.74–3.69; 0.221)
Total	459	249 (54.2)	210 (45.8 [41.2–50.3])	—	—	—	—	—

*Note*: Bold type identifies the variable‐level summary row for each variable and does not denote statistical significance.

Abbreviaions: CI, confidence interval; GEE, generalised estimating equations; OR, odds ratio; aOR, adjusted odds ratio.

^a^Unadjusted odds ratios from patient‐clustered GEE models containing each variable alone.

^b^Model A, multivariable GEE model containing practice sector, patient age, gender, age of restoration and post retention design.

^c^Model B, multivariable GEE model containing operator qualification, patient age, gender, age of restoration and post retention design. Practice sector and operator qualification were structurally collinear (Cramér’s *V* = 0.663, *p*  < 0.001) and were therefore not entered simultaneously; dashes denote the variable omitted from that model. Patient age was entered in both multivariable models as a continuous predictor, so the adjusted odds ratio is reported per year of age on the variable row and dashes appear against the individual decade bands.

‖Patient‐level characteristic tabulated at the tooth level; patient‐level distributions are reported in the Section 3. Fisher–Freeman–Halton exact test, applied because expected cell counts were below five in the Royal Medical Services category (*n* = 7).

^b^Model A, multivariable GEE model containing practice sector, patient age, gender, age of restoration and post retention design.

^∗^Pearson *χ* ^2^ test unless otherwise indicated.

In multivariable GEE Model A (evaluating the practice sector alongside patient age, gender, restoration age and post‐design; Table 3), satisfactory technical quality remained independently associated with treatment in university faculty/specialty clinics (aOR = 3.76, 95% CI: 1.95–7.23, *p*  < 0.001) and university student clinics (aOR = 2.05, 95% CI: 1.11–3.77, *p* = 0.022) compared with the private sector, passive post‐design (aOR = 1.89, 95% CI: 1.16–3.08, *p* = 0.011), and younger patient age (aOR = 0.97 per year of age, 95% CI: 0.96–0.99, *p* = 0.001). In multivariable GEE Model B (evaluating operator qualification alongside patient age, gender, restoration age and post‐design; Table 3), passive post‐design remained strongly and independently associated with satisfactory quality (aOR = 2.86, 95% CI: 1.78–4.61, *p*  < 0.001), whereas operator qualification categories were no longer statistically significant (undergraduate aOR = 0.98, *p* = 0.961; postgraduate aOR = 1.36, *p* = 0.425; specialist aOR = 1.18, *p* = 0.552 vs. general practitioner; overall Wald *χ* ^2^ = 0.791, df = 3, *p* = 0.852). This indicates that the apparent technical advantage of student operators was attributable to the institutional use of passive post systems rather than to operator seniority per se. Estimates for the Royal Medical Services setting rest on seven teeth and are correspondingly imprecise (57.1% satisfactory, 95% CI: 25.0%–84.2%; aOR = 2.47, 95% CI: 0.37–16.54); this category is retained for completeness but no inference is drawn from it. After multivariable adjustment, neither age of restoration (overall adjusted Wald *χ* ^2^ = 5.546, df = 2, *p* = 0.062) nor gender (*p* = 0.170) was independently associated with technical quality.

## 4. Discussion

This multicentre retrospective cross‐sectional study evaluated the radiographic technical quality and periapical status of 459 post‐retained restorations in 366 patients across different clinical practice sectors in Jordan, examining how endodontic, restorative, patient and operator variables were associated with these findings after accounting for intra‐patient clustering and confounding.

The predominance of post‐retained restorations in maxillary teeth in the present sample (68.6%) aligns closely with Albashaireh et al. [2], who reported that 72.7% of post‐retained restorations were placed in the maxilla. Similarly, Al‐Hamad et al. [9] observed maxillary anterior teeth to be the most frequently restored group (38.8%), comparable to the 32.9% of maxillary anterior restorations recorded in our cohort. This distribution likely reflects the higher susceptibility of maxillary anterior teeth to dental trauma and aesthetic demand, alongside the substantial loss of coronal structure commonly encountered in endodontically treated premolars, which represented the single most frequently restored tooth type in our study (46.8%).

Establishing universal criteria for optimal intraradicular post length remains a subject of clinical debate [28]. Classical prosthodontic guidelines have variously recommended a post length equal to the clinical crown [10, 29], half the root length [30], midway between the radiographic apex and the alveolar crest [31], retaining 4–5 mm of apical gutta‐percha [22–27] or extending to two‐thirds of the root length [32]. Traditional conventions emphasise that the post length should at least equal clinical crown height to optimise retention and distribute functional stresses [7, 10, 21]. In the present study, only 33.8% of restorations met this ≥ 1:1 post‐to‐crown benchmark, while 16.6% exhibited a ratio below 0.5:1—findings nearly identical to earlier audits reporting adequate post‐to‐crown length in approximately one‐third of cases [7, 8]. Notably, in our multivariable GEE analysis of periapical status, PAR exhibited a significant progressive gradient across post‐to‐crown ratio categories (21.3% for ≥ 1:1, 33.8% for 0.5–<1:1, and 46.1% for <0.5:1), with both <0.5:1 (aOR = 2.87, *p* = 0.003) and 0.5–<1:1 (aOR = 1.90, *p* = 0.020) being independently associated with higher odds of PAR. Short posts may reflect inadequate canal negotiation or conservative post‐space preparation in curved or obliterated canals, and mechanically they may offer reduced resistance to rotational and lateral occlusal forces [21]. However, traditional geometric rules must also be contextualised against contemporary adhesive and biomechanical restorative principles. Recent three‐dimensional finite element and biomechanical analyses demonstrate that stress distribution, cuspal deflection and load transfer in restored teeth depend critically on the interplay between restorative material elastic modulus, polymerisation shrinkage kinematics, luting interface integrity and structural cavity configuration rather than post‐length alone [33]. Furthermore, clinical prognosis is heavily governed by the presence of a 1.5–2.0 mm coronal ferrule and the volume of remaining coronal dentine [1–4], parameters that cannot be fully quantified on two‐dimensional periapical radiographs.

When post‐length was expressed relative to root length, 61.2% of teeth had a post‐to‐root ratio below 0.5:1, 25.3% ranged between 0.5:1 and 0.65:1, and 13.5% exceeded 0.65:1, mirroring the distribution reported by Jamani et al. [8] (57.1% <0.5, 39.3% between 0.5 and 0.66, and 3.2% >0.66). Passive posts accounted for 57.1% of restorations, whereas 42.9% were active threaded posts—predominantly placed in private practice settings (75.0% active). Although active posts provide high mechanical retention in short roots, classic literature indicates that engaged threads can introduce internal wedging stresses during insertion and functional loading [1]. When post retention design was entered into our multivariable GEE models for technical quality, passive posts demonstrated significantly higher adjusted odds of satisfactory technical quality in both multivariable models (aOR = 1.89, 95% CI: 1.16–3.08, *p* = 0.011 in Model A; aOR = 2.86, 95% CI: 1.78–4.61, *p*  < 0.001 in Model B) compared with active posts, mediating the apparent differences previously attributed to operator qualification. Post‐retention design was not, however, associated with periapical status in the adjusted model (aOR = 1.04, 95% CI: 0.62–1.74, *p* = 0.890). Root fracture was recorded in 2.0% of teeth (eight of 197 active posts and one of 262 passive posts) and post‐fracture in 0.7%; these events were too infrequent for formal analysis by post‐design, and no association between post type and fracture was tested in the present study.

Preserving an adequate apical segment of root canal filling material during post‐space preparation is widely advocated to prevent microbial coronal‐to‐apical percolation. Published recommendations range from a minimum of 3 to 5 mm [7, 9, 34, 35], with 4–5 mm most commonly regarded as ideal [22–27]. In our sample, 60.6% of teeth retained >5.0 mm of gutta‐percha, 27.0% retained 3.0–5.0 mm (including 17.6% with 4.0–5.0 mm), 5.9% retained <3.0 mm, and 6.5% (*n* = 30) showed no radiographically detectable gutta‐percha apical to the post tip. Within the limitations of two‐dimensional radiographic assessment, teeth with no radiographically detectable gutta‐percha apical to the post exhibited the highest prevalence of PAR (53.3%), whereas PAR prevalence did not differ significantly across the categories in which gutta‐percha was retained (*p* = 0.857). Because a two‐dimensional radiograph cannot directly measure microbiological leakage or three‐dimensional sealing ability, these findings should be interpreted strictly as a radiographic association: among the obturation variables examined, absence of radiographically evident root filling and overextension beyond the apex showed the strongest associations with PAR.

Axial deviation of the post in the mesio‐distal plane (>2°) was identified in 57 teeth (12.4%), with measurable angulation logged in 50 teeth (mean: 10.4° ± 6.1°), falling between lower rates of 2.3% [9] and higher rates of 20%–25% [7, 8] reported in previous radiographic surveys. Careful selection of heated pluggers or non‐end‐cutting rotary Gates‐Glidden and Peeso drills following canal curvature is recommended to minimise axial deviation and root perforation [36], which was observed in 1.3% (*n* = 6) of teeth in the present cohort.

Apical termination of the root canal filling within 0–2.0 mm of the radiographic apex is a well‐established benchmark of endodontic technical quality [18, 19]. In this study, 69.3% of teeth demonstrated adequate root filling length, comparing favourably with earlier reports of ~61% [5, 19], while 19.2% were underfilled (>2 mm short), 5.4% were overextended, and 6.1% lacked any radiographic root filling [5, 8, 9, 37]. A radiolucent gap between the post tip and the residual root filling was present in 30.9% of teeth (mean 1.34 ± 1.02 mm across the 138 of 142 gaps for which a measurement was recorded), unsatisfactory lateral post adaptation was noted in 21.6%, and defective coronal margins were present in 36.8% [38]. While poor coronal marginal adaptation (OR = 1.74, *p* = 0.010), poor obturation density (OR = 1.82, *p* = 0.006), and secondary caries (OR = 2.22, *p* = 0.006) were each significantly associated with PAR in univariable analyses, multivariable GEE modelling demonstrated that absent root fillings (aOR = 3.09, *p* = 0.014) and overextended root fillings (aOR = 3.00, *p* = 0.018) were the primary endodontic predictors of PAR.

Overall, PAR was detected in 31.6% of evaluated teeth, within the 16%–53.9% range reported in prior cross‐sectional populations [7–9, 24]. For comparison, Kvist et al. [24] recorded PAR in 16% of posted roots and 13% of roots without posts, appreciably below the prevalence observed here, although differences in population, era and unit of assessment limit direct comparison. Across root filling length categories, PAR prevalence exhibited a significant linear trend (*p* = 0.002), increasing from 28.0% in adequately filled canals to 44.0% in overextended fillings and 53.6% in canals with no radiographic filling. Nevertheless, 61.4% (89/145) of all teeth presenting with PAR had an adequate radiographic root filling length (0–2 mm short of the apex), underscoring that two‐dimensional radiographic length alone does not guarantee periapical health. In addition to persistent intraradicular or extraradicular infection and inadequate chemomechanical debridement, contemporary evidence highlights the biological and cytotoxic profile of endodontic root canal sealers in contact with periradicular tissues—particularly when extruded beyond the apical foramen—as an important determinant of periapical tissue response and delayed healing [39].

Complications associated with post‐retained restorations range from restorable defects to catastrophic failures requiring extraction [40]. In the present study, fewer than half of the evaluated restorations (45.8%) met the criteria for satisfactory core technical quality. Importantly, our multivariable GEE analyses revealed several critical insights into operator‐, sector‐, post‐design and time‐related variables. First, while unadjusted comparisons suggested that older restorations (>5 years) had a higher prevalence of unsatisfactory radiographic findings than restorations placed within <1 year (65.5% vs. 44.8%, *p* = 0.042), this association was no longer significant after multivariable adjustment (overall adjusted Wald *χ* ^2^ = 5.546, df = 2, *p* = 0.062). This indicates that the unadjusted difference reflected a higher proportion of older restorations sampled from the private sector rather than longitudinal deterioration over time. Second, regarding operator qualification, undergraduate dental students (59.2% satisfactory) and postgraduate Master’s students (53.3% satisfactory) achieved higher unadjusted satisfactory rates than general practitioners (34.5%) and specialists (46.1%). However, adjusting for post‐design in multivariable Model B completely eliminated the independent effect of operator qualification, demonstrating that the apparent advantage of student operators was driven by the strict institutional use of passive post systems (95.9% passive in undergraduate clinics vs. 78.4% active in general practice) alongside mandatory step‐by‐step faculty supervision. Meanwhile, practice sector remained an independent predictor of quality in Model A (aOR = 3.76 for university specialty clinics and 2.05 for student clinics vs. private practice, *p*  < 0.05). Taken together, these findings emphasise that clinical decision‐making and quality assurance should integrate endodontic obturation quality, preservation of apical filling material, axial post alignment, coronal marginal adaptation and the selection of passive post systems rather than relying on any single geometric ratio.

### 4.1. Study Limitations

Several limitations should be considered. The retrospective cross‐sectional design captures a single time point without baseline radiographs or treatment chronologies, so causal and prognostic inferences cannot be drawn and PARs (PAI ≥ 3) cannot be distinguished between persistent, healing and scar lesions. Clinical determinants of prognosis—ferrule dimensions, remaining coronal dentine, periodontal status, occlusal loading and symptoms—cannot be assessed radiographically, and two‐dimensional projection precludes evaluation of buccolingual post‐deviation and may obscure minor voids, lateral perforations or root fractures. Acquisition was heterogeneous, combining institutional digital PSP images with external films digitised at 600 dpi; despite individual calibration, dimensionless post‐ratios and standardised contrast adjustment, differences in resolution and projection distortion may have introduced measurement or classification error, and blinding extended to patient identity but not to treatment centre. In multi‐rooted teeth (16.1%), measurements were restricted to the primary post in the widest canal, whereas PAI was scored at tooth level, a potential canal‐to‐tooth mismatch, although this root‐level approach follows established practice in this literature [24]. Intra‐patient clustering was modelled using GEE, but external records carried no clinician identifiers, so operator‐level clustering could not be addressed and residual confounding by unmeasured case complexity or referral pattern cannot be excluded. Finally, the participating private practices and the single Royal Medical Services centre were not randomly selected, so the sample is complete within the settings studied rather than representative of post‐retained restorations in Jordan generally; any post not entered under the relevant procedure code would not have been retrieved; and the composite classification was study‐specific, with item‐level reliability statistics not archived during data collection.

## 5. Conclusions

Within the limitations of this multicentre retrospective cross‐sectional study, fewer than half (45.8%) of evaluated post‐retained restorations demonstrated satisfactory radiographic technical quality. Tapered, prefabricated posts‐predominated, and while 69.3% of teeth exhibited an adequate root canal filling length, only 33.8% achieved a post‐to‐crown length ratio ≥ 1:1. PAR (PAI ≥ 3) was detected in 31.6% of teeth and was independently associated in multivariable GEE analysis with absent or overextended root canal fillings, lower post‐to‐crown length ratios (<0.5:1 and 0.5–<1:1), and anterior or premolar tooth types. After adjusting for intra‐patient clustering and confounding, satisfactory technical quality was independently associated with treatment in university faculty specialty and student clinics compared with the private sector, passive post‐design and younger patient age, whereas operator qualification (after controlling for post‐design) and restoration age were not independently associated with radiographic quality.

## Author Contributions

**Khawlah Albashaireh:** conceptualisation, methodology, investigation, data collection, data analysis, writing – original draft. **Ghada Maghaireh:** investigation, validation, writing – review and editing. **Maram Jaradat:** formal analysis, writing – review and editing. **Zakereyya Albashaireh:** conceptualisation, methodology, data collection, review and editing.

## Funding

This research received no specific grant from any funding agency in the public, commercial or not‐for‐profit sectors.

## Disclosure

All authors have read and approved the final manuscript.

## Ethics Statement

The study protocol received formal ethical approval from the Institutional Review Board of Jordan University of Science and Technology (IRB No. July2025/184‐5). Given the retrospective nature of the study, the requirement for informed consent was waived.

## Conflicts of Interest

The authors declare no conflicts of interest.

## Data Availability

The data supporting the findings of this study are available from the corresponding author upon reasonable request.
